# micro-RNAs dependent regulation of DNMT and HIF1α gene expression in thrombotic disorders

**DOI:** 10.1038/s41598-018-38057-6

**Published:** 2019-03-20

**Authors:** Aatira Vijay, Prabhash Kumar Jha, Iti Garg, Manish Sharma, Mohammad Zahid Ashraf, Bhuvnesh Kumar

**Affiliations:** 1 Defence Institute of Physiology and Allied Sciences, DRDO, Delhi, India; 2 Department of Biotechnology, Jamia Millia Islamia, Delhi, India

## Abstract

MicroRNAs (miRNAs) are involved in a wide variety of cellular processes and post-transcriptionally regulate several mechanism and diseases. However, contribution of miRNAs functioning during hypoxia and DNA methylation together is less understood. The current study was aimed to find a shared miRNAs signature upstream to hypoxia (via HIF gene family members) and methylation (via DNMT gene family members). This was followed by the global validation of the hypoxia related miRNA signature using miRNA microarray meta-analysis of the hypoxia induced human samples. We further concluded the study by looking into thrombosis related terms and pathways enriched during protein-protein interaction (PPI) network analysis of these two sets of gene family. Network prioritization of these shared miRNAs reveals miR-129, miR-19band miR-23b as top regulatory miRNAs. A comprehensive meta-analysis of microarray datasets of hypoxia samples revealed 29 differentially expressed miRNAs. GSEA of the interacting genes in the DNMT-HIF PPI network indicated thrombosis associated pathways including “Hemostasis”, “TPO signaling pathway” and “angiogenesis”. Interestingly, the study has generated a novel database of candidate miRNA signatures shared between hypoxia and methylation, and their relation to thrombotic pathways, which might aid in the development of potential therapeutic biomarkers.

## Introduction

Venous thromboembolism (VTE) is the third most fatal cardiovascular complication. Around one in every three patients with VTE, develops pulmonary embolism (PE)-a condition in which thrombus drifts through the blood flow and gets lodged into the lungs; two in every three patients manifest deep vein thrombosis (DVT)^1^. The etiology of thrombosis is multifactorial; both inherited and acquired factors contribute towards the pathophysiology of thrombosis.Apart from the classical factors, hypoxic exposure, as experienced during high altitude sojourning, long term air travel, as well as, during blood flow restriction and heavy exercise, has been proposed as a new attribute to increase the likelihood of developing thrombosis^2^. Further, gene expression profiling has suggested multiple genes involved in the regulation of VTE during environmental stress conditions, like high altitude hypoxia^3^.

Epigenetic modifications, specifically DNA methylation is associated with magnitude of diseases^4^. Aberrant DNA methylation has an impact on promoter accessibility and gene expression and has been linked to diseases previously. A recent study has observed the regulation of DNA methyltransferases (DNMTs) - key enzymes catalyzing methylation, by HIF-1α, which is the cellular mediator of hypoxia and only observed in the hypoxic stress conditions. Mutated HIF1α binding site significantly reduced the promoter activity in DNMT enzymes, indicating that hypoxia activates DNMTs via HIF response element (HRE), identified in the promoter of DNMT1 and DNMT3B^5^. MicroRNAs are noncoding endogenous RNA molecules of 21–25 nucleotides long and are involved in non-classical post transcription modifications. It is suggested that DNMT1is regulated via miRNAs in human malignant cholangiocytes^6^. Also, the regulation of DNMTs in human aortic cells via miRNAs in hypermethylation has been discussed previously, where the down-regulation of miRNA-152 led to increased DNMT activity resulting in silencing of Estrogen receptor α gene^7^. While the role of DNMTs-miRNAs, HIF1α-miRNAs, and HIF1α-DNMTs association has been appreciated individually, the effect of these associations and their cumulative effect on the coagulation cascade followed by thrombotic complications have not been completely understood.

miRNAs play crucial role in the regulation of biological functions like cell division, differentiation, signaling and death, and dysregulation of miRNAs may lead to many diseases^8,9^. miRNAs have a number of targets, similarly, one gene may act as a target for multiple miRNAs. Here, in this study we proposed putative miRNA binding site on DNMT and HIF family genes, suggesting the role of miRNAs in regulating gene expression of these families. The online available tools like miRDB, Targetscan, RNA22 etc. were employed. We sought to investigate the shared miRNAs by these two families and further predicted the targets of those common miRNAs. The predictions done by these tools are based on miRNA-mRNA binding via seed sequence, accessibility of the target gene, free energy of the miRNA-mRNA hybrid and conservation across the species^10^. Since multiple algorithms are used to predict the shared miRNAs and the target genes for candidate miRNAs as well, it is often observed that prediction from different softwares is dissimilar^11^; therefore, it is perilous to validate the findings by different tools.

Epigenetic modifications and changes in the epigenetic machinery occur during hypoxic exposure and this may contribute to pathophysiological events. Among many other studies, one of the reports suggests increased hypoxia in tumours increases hypermethylation, while restoration of tumour oxygenation abrogates this effect. Tumour hypoxia, therefore, acts as a novel regulator of DNA methylation^12^. Besides, there are an array of articles published establishing the links between miRNA and DNA methylation^13–15^; miRNA and hypoxia^16,17^. However, there is little known about the regulatory role of miRNA in linking these two physiological events, therefore, we intended to design our study to look for the pool of shared miRNAs via the available databases of miRNA and to find out the putative miRNAs interacting the main players of methylation (viz. DNMT1, DNMT3A and DNMT3B) and hypoxia (viz. HIF1A, EPAS1, HIF3A, ARNT and ARNT2). This was followed by the global validation of the putative interaction between hypoxia and miRNAs by performing the miRNA microarray meta-analysis of the hypoxia exposed experimental gene expression data available on public platforms (GEO, ArrayExpress etc.). The objective of this study was to identify an association between hypoxia and DNA methylation together via an upstream common regulator-miRNA, and their impact on thrombotic pathways and genes. The probability of these factors acting in concert and regulated via a common set of miRNAs based mechanism is suggested.

## Results

**Figure 1:**
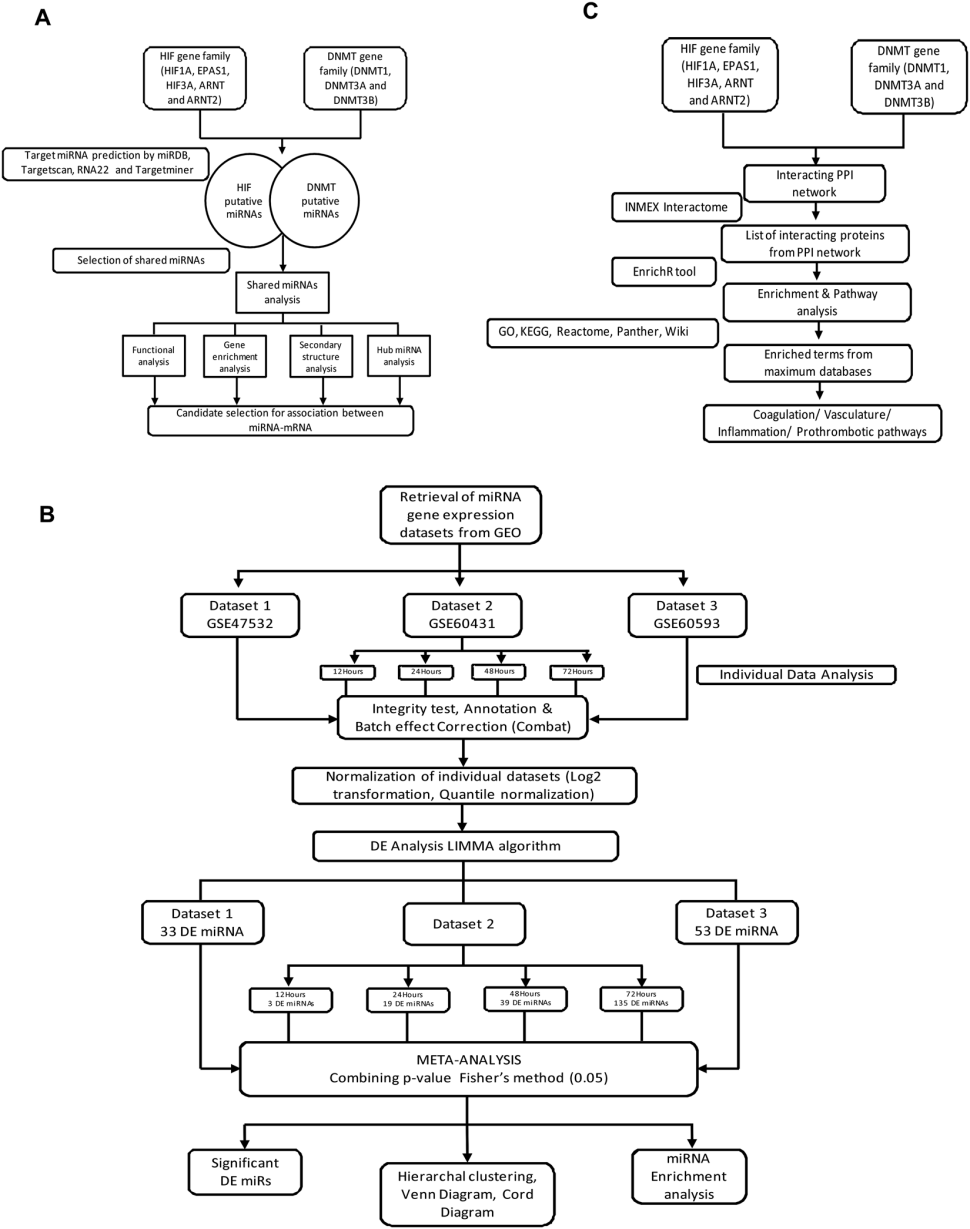
Workflow of analysis strategy. (**A**) Phase I- Workflow depicting the analysis strategy applied to uncover the shared miRNA candidate between HIF and DNMT gene families and subsequent analysis of the candidate miRNAs. (**B**) Phase II- Depiction of the flow chart of the process involved in global validation of phase I by integrated meta-analysis of the selected miRNA microarray datasets from the hypoxia exposed samples from human studies. (**C**) Phase III- Workflow depicting the analysis strategy for Interacting PPI network analysis of DNMT and HIF family genes to discern their association with thrombosis.

The study incorporates three phase analysis (strategy detailed in Fig. 1) which resulted in linking shared signature of miRNA between hypoxia and methylation, followed by intrinsic biological validation of the hypoxia-miRNA axis and finally relating its association with thrombotic terms/pathways.

### Analysis of shared miRNA between HIF and DNMT gene family reveals pathways associated with regulation of gene expression and pathways related to vasculature

**Figure 2:**
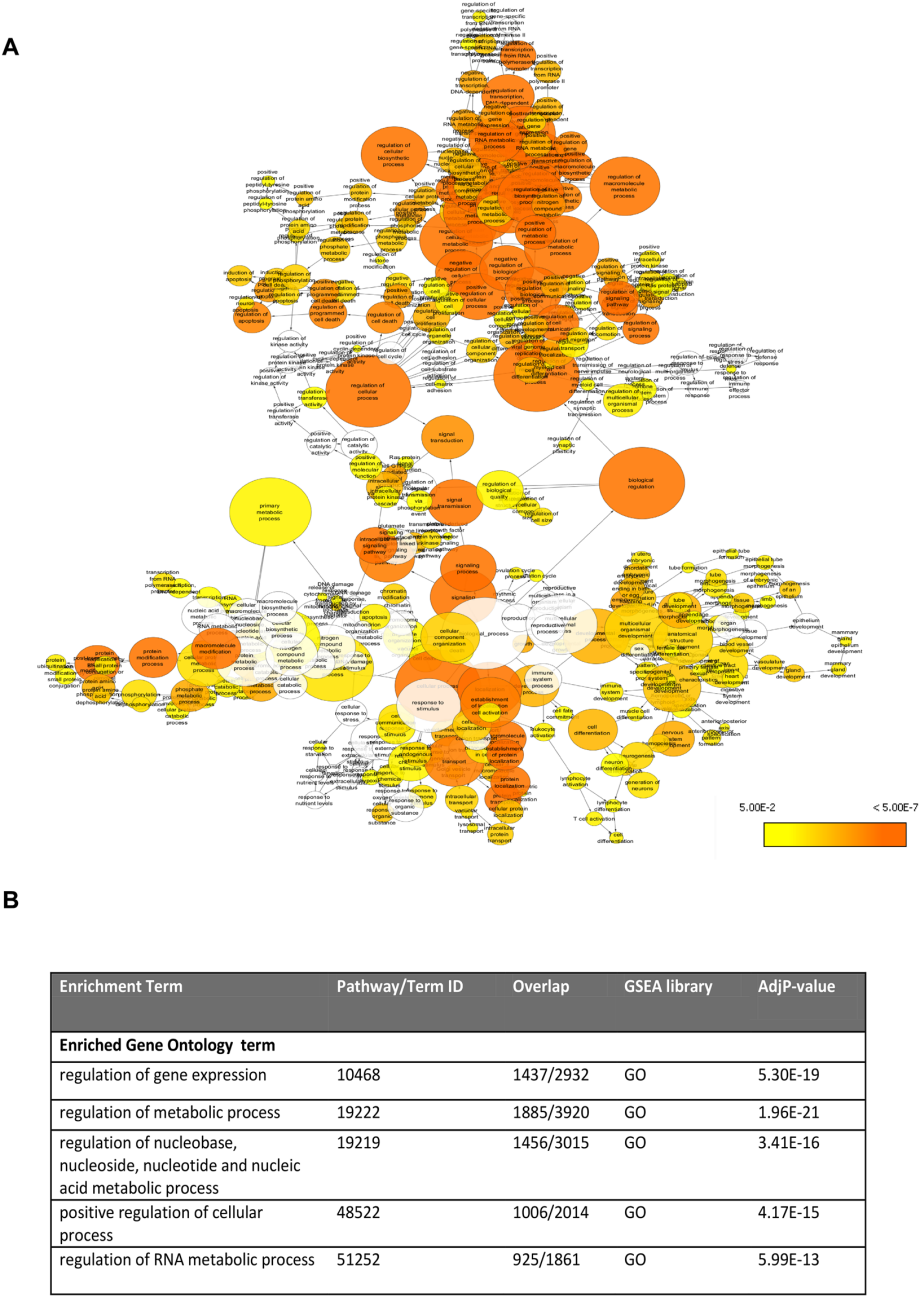
Overrepresentation of Gene Ontology categories in Biological Networks identified from the list of target genes of shared miRNAs between HIF and DNMT family. (**A**) Enrichment network of shared DEGs based on biological processes. Significantly overrepresented biological processes based on GO terms were visualized in Cytoscape. The size of a node is proportional to the number of targets in the GO category. The color represents enrichment significance— the deeper the color on a color scale, the higher the enrichment significance. P-values were adjusted using a Benjamini and Hochberg False Discovery Rate (FDR) correction. (**B**) Table showing top five GO terms associated with the target genes. Overlap: indicates the number of hits from the meta-analysis compared to each curated gene set library; GO: gene ontology biological process.

Analysis of the shared miRNAs between the two gene families (HIF and DNMT) indicates regulatory relationships at the level of gene expression. Since the *in silico* prediction tools have their own set of pros and cons, we did the shared miRNA selection and its target prediction using five different programs in the present study to ensure high specificity in target prediction. Based on the conservation efficiency, we curated a list of 30 high confidence miRNAs shared between HIF and DNMT gene family, some of the important shared miRNAs obtained from our analysis are miR-29, miR-23 and miR-19 (Supplementary Table 1 and Supplementary Sheet 1). Target prediction of these 30 shared miRNAs resulted in a list of thousands of target genes. We further generated a compact list of target genes for GSEA based on seed score and duplicate removal. To explore potential functional roles of these prognostic shared miRNAs in regulation of hypoxia (HIF) and methylation (DNMT) pointing towards thrombotic association, we performed functional enrichment analysis of their associated target genes. Functional enrichment revealed prognostic role of regulation of gene expression (GO:10468), regulation of metabolic process (GO:19222) and regulation of RNA metabolic process (GO:51252) etc. (Fig. 2 & Supplementary Sheet 2). GSEA analysis of target genes for shared miRNAs was done to investigate whether they correlate with thrombogenic pathways; in this analysis using various databases classification, functionally related pathway terms could be associated with vasculature alteration which includes- Signaling events mediated by VEGFR1 and VEGFR2 (NCI-NATURE), EGF receptor signaling pathway (Panther), Endothelin signaling pathway (Panther) and NFAT and Hypertrophy of the heart (Biocarta) etc. among the top ten significant pathways (Supplementary Table 2).

### Network biology prioritization of shared miRNAs reveals miR-129, miR-19b and miR-23b as the top regulatory miRNAs

**Figure 3:**
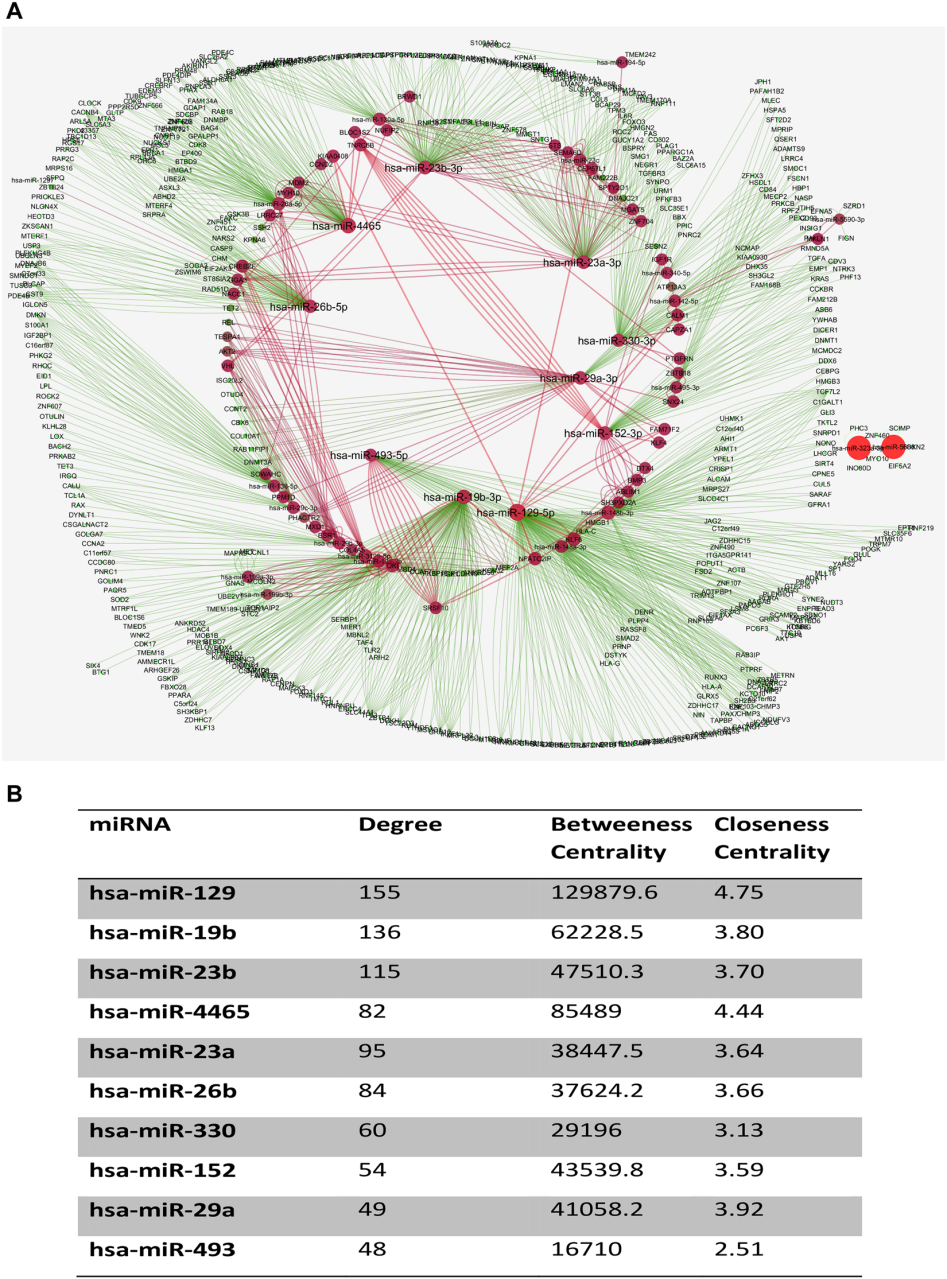
Hub shared miRNA and candidate prioritization. (**A**) Interaction network between shared miRNAs and target genes to perform hub miRNA analysis based on complex network centrality measures. The source nodes here are the miRNAs and target nodes are coding mRNAs (target genes), while the edges represent the interaction between them. The size and color of the nodes in network is relative to the betweenness centrality. The top hub miRNAs in the network are highlighted with bigger font size. (**B**) List of top hub miRNA selected from in silico analysis of miRNAs-mRNAs network based on scoring algorithm.

Based on the shared binding patterns of the miRNAs in the HIF and DNMT family genes, we hypothesized that these miRNAs might be involved in thrombosis development and influence its progression. Thus, we tested the efficiency of the hub miRNAs as regulatory signatures for hypoxia regulated methylation leading to thrombosis. In biological networks, hubs are commonly defined as the top nodes showing maximum number of interactions with other nodes in the network defined as degree, apart from this, other centrality parameters of network viz. Betweenness and closeness centrality were considered for selecting the hub miRNAs. Consequently, a coexpression network was constructed that included the shared miRNAs and target genes. We used validated interaction between the shared miRNA and its target genes from miRTarbase database and drew the network using Cytoscape (Fig. 3A). Our data showed that the network was composed of 30 source nodes (miRNAs) and 1512 target nodes (target mRNAs). This hub network indicated that one miRNA could target, at most, 155 coding genes. After performing the network analysis of the miRNA-target gene network, top ten hub miRNAs were selected based on the complex centrality parameters (Fig. 3B). Among the top hub miRNAs were miR-129 (degree = 155; BC = 129889.6 and CC = 4.75), miR-19b (degree = 136; BC = 62228.5 and CC = 3.80) and miR-23b (degree = 115; BC = 47510.3 and CC = 3.70) as detailed in Fig. 3.

### DNMT3A and HIF1A shows binding sites for several cardiovascular disorder associated miRNAs

BLAST alignment analysis was performed for DNMT3A and HIF1A with the mature miRNA of human from the Targetscan database, and the results showed hundreds of interactions between these two genes and miRNA (Supplementary Sheet 1). The list of interaction was used to construct a target gene-miRNA network (Fig. 3A) resulting in hsa-miR-129, miR-19b and hsa-miR-330 being the top interacting miRNAs with 155, 136 and 60 interaction each with different target genes (Fig. 3B). The miRNA binding sites on the DNMT3A and HIF1A genes were predicted using a web-based program RNAhybrid (version: 2.2) by using the FASTA sequence of these genes and the mature sequence of its binding miRNAs. We also calculated the miRSVR score of the mRNA-miRNA interaction using the microRNA.org tool. Based on the minimum free energy (MFE), most stable interaction were found to be between DNMT3A-miR129 with MFE = −27.9 kcal/mol and miRSVR score = −0.645, DNMT3A-miR19b with MFE = −25.8 kcal/mol and miRSVR score = −0.104, HIF1A-miR19b with MFE = −21.5 kcal/mol and miRSVR score = −0.147, and HIF1A-miR330 with MFE = −26.1 kcal/mol and miRSVR score = −0.981 respectively (Table 1). It was suggested that these key interactions may play important roles in the regulation of gene expression during hypoxia mediated methylation.

**Table 1:** RNAfold analysis of miRNA-target genes interaction.

Target Gene	Binding between target gene and miRNA	miRSVR score	Phastcons score	Min.Free Energy(kcal/mol)
miR-129-DNMT3A	3′ cguucgggucuggc**GUUUUU**c 5′ hsa-miR-129-5p | | | | | | 166:5′ caguauuuaaaaau**CAAAAA**a 3′ DNMT3A	−0.645	0.684	−27.9
miR-19b-DNMT3A	3′ agucaaaacgUACCU**AAACGU**Gu 5′ hsa-miR-19b: : | | | | | | | | : 766:5′ gcccugccagGUUGU**UUUGCA**Ua 3′ DNMT3A	−0.104	0.787	−25.8
miR-19b-HIF1A	3′ agUCAAAACGU –– ACCUA**AACGUG**u 5′ hsa-miR-19b | | | | | | | | | | | | | | 388:5′ aaAUUUUUACACCUUUUU**UUUCAC**a 3′ HIF1A	−0.147	0.671	−21.5
miR-330-HIF1A	3′ agagacguccggcac**ACGAAAC**g 5′ hsa-miR-330-3p | | | | | | | 415:5′ uuacauaaauaauaa**UGCUUUG**c 3′ HIF1A	−0.981	0.671	−26.1

Table depicting miR binding site on DNMT and HIF1A, miRVR score by miRanda target prediction tool, binding energy and secondary binding structure were calculated using RNA HYBRID tool.

### Identification of common differentially expressed miRNAs from hypoxia related miR microarray datasets

**Figure 4:**
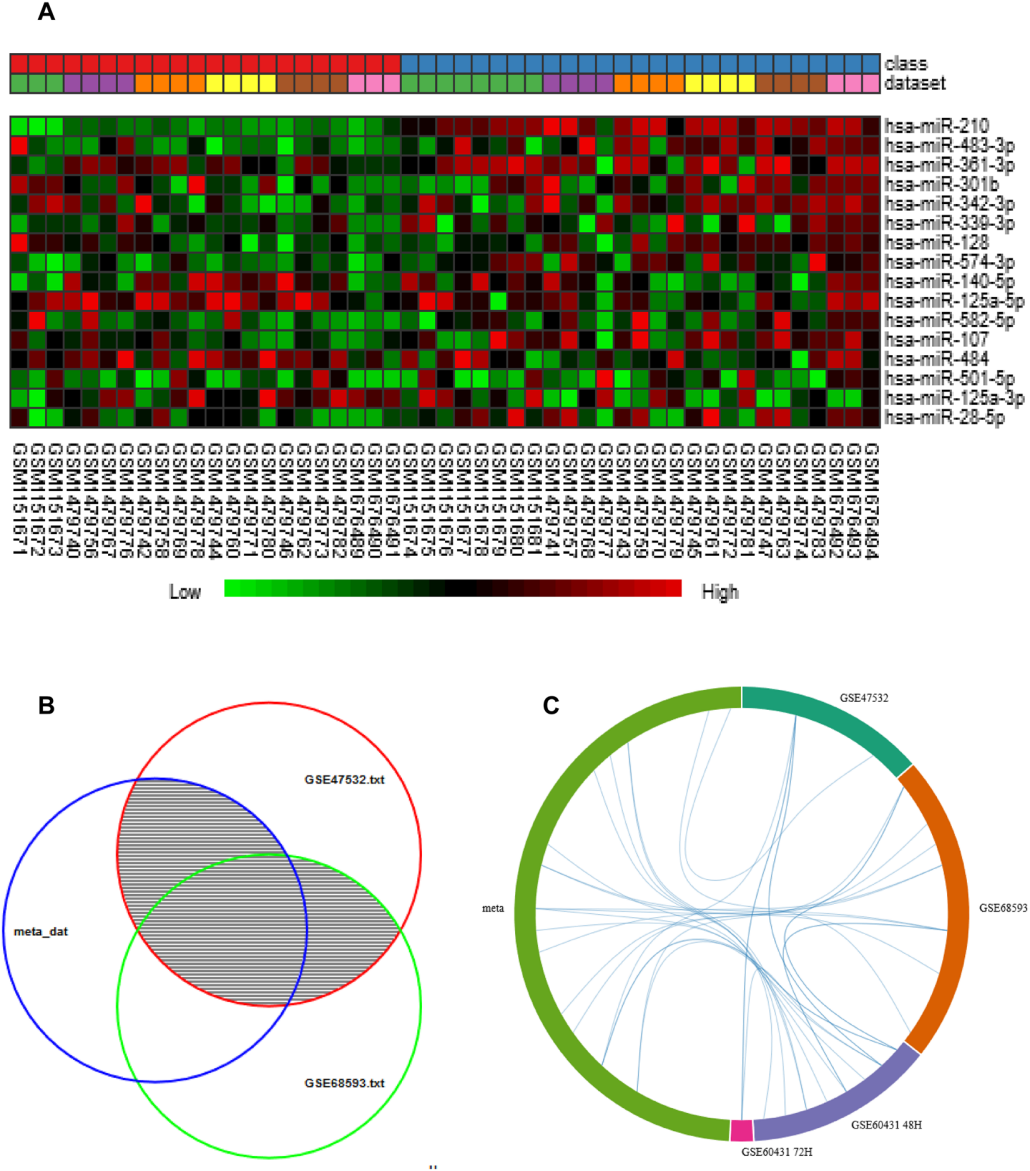
miRNA expression pattern of the hypoxia induced samples from meta-analysis. (**A**) Heat-map representation of expression profiles for the differentially expressed miRNA obtained from meta-analysis. Clustering of selected genes on the heat-map was performed by hierarchical clustering algorithm using Euclidean distance measure. Class 1 (Red): Control samples; Class 2 (Blue): Hypoxia exposed samples. (**B**) Venn diagram of differentially expressed miRNAs identified from the meta-analysis (Meta-DE) and those from each individual microarray analysis (Individual-DE). (**C**) Chord diagram showing the differentially expressed miRNAs connection between the individual datasets and the meta-data.

To identify a miRNA signature in the hypoxic condition, three microarray studies (Table 2) were analyzed using Integrative Meta-Analysis of Expression Data (INMEX), a web interface for the integrative meta-analysis. After the normalization, preprocessing and data integrity check of the individual datasets we conducted differential expression meta-analysis across hypoxia exposed and control samples using combining p-value (Fisher’s method). This method of gene expression meta-analysis is considered to be the most popular since it generates most consistent biological results. Combining p-value (Fisher’s method) has the advantage of allowing more sensitive results i.e. detecting more DE (differentially expressed) genes. Our analysis identified a total of 29 DE miRNAs including 20 overexpressed and 9 underexpressed miRNAs across the datasets under the significance threshold of p < 0.05 (Table 3). Figure 4 shows the heatmap of all the differentially expressed miRNAs across all datasets. miRNA-210 (combined Tstat = 128.6 and combined pval = 0), miRNA-483 (combined Tstat = 59.4 and combined pval = 4.47E-06) and miRNA-361 (combined Tstat = 55.0 and combined pval = 1.88E-05) were among the most significantly overexpressed genes while, miRNA-520c (combined Tstat = −52.6 and combined pval = 3.82E-05), miRNA-516a (combined Tstat = −45.0 and combined pval = 3.99E-04) and miRNA-372 (combined Tstat = −44.6 and combined pval = 3.4.17E-04) were the most underexpressed genes across the three microarray datasets (Table 3). The complete list of miRNA probes both sample wise and dataset wise along with its expression intensities is provided in Supplementary Sheet 3. The main objective of this phase was to conduct global validation of the hypoxia-miRNA axis from phase I. Therefore, when we matched the DE miRNAs with the putative miRNAs associated with HIF gene family (Supplementary Sheet 1), it resulted in matching of 25 DE miRNAs with the putative miRNAs binding with either of the five genes from hypoxia gene family (detailed in Supplementary Table S5).

**Table 2:** Characteristics of individual studies included in the miRNA meta-analysis of hypoxic exposure.

S. No.	GEO accession no.	Platform of dataset	Samples(Ctl/exp)	No. of miRNAs	Organism	Sample source	Platform	Model of generating expression summaries	Reference
1	[GSE47532]	GPL8227	(n = 11) 3/8	820	Homo Sapiens	breast cancer cell line MCF-7	Agilent-019118 Human miRNA Microarray 2.0 G4470B	log2 transformed and quantile normalized	^73^
2	[GSE60432 12 h]	GPL14550	(n = 08) 4/4	851	Homo Sapiens	human placental trophoblast cells	Agilent-028004 SurePrint G3 Human GE 8 × 60 K Microarray	log2 transformed and quantile normalized	^74^
	[GSE60432 24 h]	GPL14550	(n = 08) 4/4	851	Homo Sapiens	human placental trophoblast cells	Agilent-028004 SurePrint G3 Human GE 8 × 60 K Microarray	log2 transformed and quantile normalized	^74^
	[GSE60432 48 h]	GPL14550	(n = 08) 4/4	851	Homo Sapiens	human placental trophoblast cells	Agilent-028004 SurePrint G3 Human GE 8 × 60 K Microarray	log2 transformed and quantile normalized	^74^
	[GSE60432 72 h]	GPL14550	(n = 08) 4/4	851	Homo Sapiens	human placental trophoblast cells	Agilent-028004 SurePrint G3 Human GE 8 × 60 K Microarray	log2 transformed and quantile normalized	^74^
3	[GSE68593]	GPL20157	(n = 06) 3/3	1907	Homo Sapiens	Hepatocellular Carcinoma cells	Agilent-041686 Unrestricted Human miRNA Microarray	log2 transformed and quantile normalized	^75^

GEO: Gene Expression Omnibus; GSE 60432 was further separated into four subgroups with 4/4 control and hypoxia exposed samples at different time points detailed in method section. These four subgroups were considered as individual datasets during meta-analysis. All the datasets used in this study were generated using a common platform; Agilent Human miRNA Microarray (probe name version).

**Table 3:** Differentially expressed miRNAs identified in the meta-analysis.

miRNAs	Individual dataset fold change						Meta-analysis results	
	GSE47532	GSE68593	GSE60431 12H	GSE60431 24H	GSE60431 48H	GSE60431 72H	CombinedTstat	CombinedPval
**Up-regulated**								
hsa-miR-210	0.45351	0.12816	0.607	1.246	2.4248	1.0887	128.68	0
hsa-miR-483-3p	−0.02947	0.19988	0.076	0.25325	0.60175	0.25779	59.498	4.47E-06
hsa-miR-361-3p	0.13963	6.0635	−0.0855	−0.0035	0.0765	0.10783	55.075	1.88E-05
hsa-miR-301b	−0.0681	5.7552	−0.014	0.002	0.06125	0.18765	51.1	5.74E-05
hsa-miR-342-3p	−0.01063	0.12554	−0.02675	0.1645	0.56225	0.3065	46.993	2.50E-04
hsa-miR-520d-3p	−0.0484	0	0.09075	0.11675	0.35225	0.073395	46.166	2.98E-04
hsa-miR-339-3p	0.058974	5.5948	−0.02925	0.02625	0.05675	0.002168	42.961	6.69E-04
hsa-miR-574-3p	0.13376	0.11681	0.416	0.47175	0.57675	0.23595	37.716	0.003927
hsa-miR-128	−0.03101	0.092111	−0.07125	0.11725	0.4125	0.22575	37.586	0.003927
hsa-miR-520h	0.041378	0	0.1005	0.0915	0.36925	0.098232	37.526	0.003927
hsa-miR-519d	−0.01888	0	0.0715	0.241	0.321	0.063669	36.298	0.0051746
hsa-miR-582-5p	−0.07989	0.44819	−0.01475	0.10575	−0.01625	0.11772	35.7	0.0061153
hsa-miR-107	8.40E-04	0.040283	0.00775	0.04875	0.38475	0.10323	34.798	0.0080929
hsa-miR-193a-5p	0.10122	0	0.06075	0.16125	0.25325	0.13022	34.211	0.0095495
hsa-miR-516a-5p	−0.0164	0	0.0855	0.1635	0.28775	0.032626	33.268	0.012717
hsa-miR-34c-5p	0.066224	0	0.19575	0.121	0.6915	0.53017	32.327	0.016476
hsa-miR-501-5p	−0.03599	5.5245	0.30375	−0.031	0.265	−0.17533	32.068	0.01698
hsa-miR-518b	−0.00666	0	0.08675	0.279	0.14625	0.060192	32.017	0.01698
hsa-miR-887	0.22306	0	0.07125	0.271	0.63	−0.04647	30.733	0.0248
hsa-miR-28-5p	0.01494	0.17883	0.083	−0.03475	0.09975	0.13843	29.871	0.032319
**Down-regulated**								
hsa-miR-125a-3p	0.060067	−0.78435	−0.17125	−0.0925	−0.30975	−0.19433	−30.783	0.0248
hsa-miR-484	0.001933	0.26227	−0.166	−0.038	−0.26975	−0.16247	−33.168	0.012717
hsa-miR-140-5p	0.089248	0.15487	0.0465	−0.482	−1.1058	−0.32789	−36.369	0.0051746
hsa-miR-125a-5p	−0.0072	0.21208	−0.381	−0.9675	−0.83275	−0.12906	−36.373	0.0051746
hsa-miR-520b	0.04912	0	−0.239	−0.2915	−0.4175	−0.11017	−41.262	0.0011851
hsa-miR-520f	−0.02961	0	−0.32775	−0.4665	−0.74525	−0.13725	−43.872	5.15E-04
hsa-miR-372	0.035251	0	−0.26925	−0.71425	−1.1938	−0.37945	−44.681	4.17E-04
hsa-miR-516a-3p	−0.00785	0	−0.26275	−0.36675	−0.75625	−0.23978	−45.089	3.99E-04
hsa-miR-520c-3p	0.017946	0	−0.354	−0.666	−0.80975	−0.10691	−52.649	3.82E-05

Genes were ranked based according to the combined Tstat. List was generated by combining p-val (Fisher’s method) combination which takes into consideration both the direction and magnitude of gene expression changes. The table shows expression value of particular DE miRNA in both the individual data and the meta-analysis results.

### Enrichment analysis of DE miRNAs in hypoxia resulted in integrin mediated cell adhesion pathway

We performed the enrichment analysis of DE miRNAs using miRNA Enrichment Analysis and Annotation tool (miEAA), which is a web-based application that offers a variety of commonly applied statistical tests, such as over-representation analysis and miRNA set enrichment analysis, which are similar to Gene Set Enrichment Analysis. DE miRNAs in meta-analysis of hypoxia datasets results were associated with the Kyoto Encyclopedia of Genes (KEGG) pathways (P < 0.05), including “Neurotrophin signaling pathway (hsa04722)”, “Integrin mediated cell adhesion (WP185)” and “MAPK signaling pathway (WP382)”. The most important disease terms associated with DE miRNAs included “dilated cardiomyopathy (p-val = 0.015)”, colon cancer (p-val = 0.012)” and “glioma (p-val = 0.012)” (Supplementary Table 3).

### PPI network analysis of DNMT and HIF gene family reveals interacting intermediate genes network

**Figure 5:**
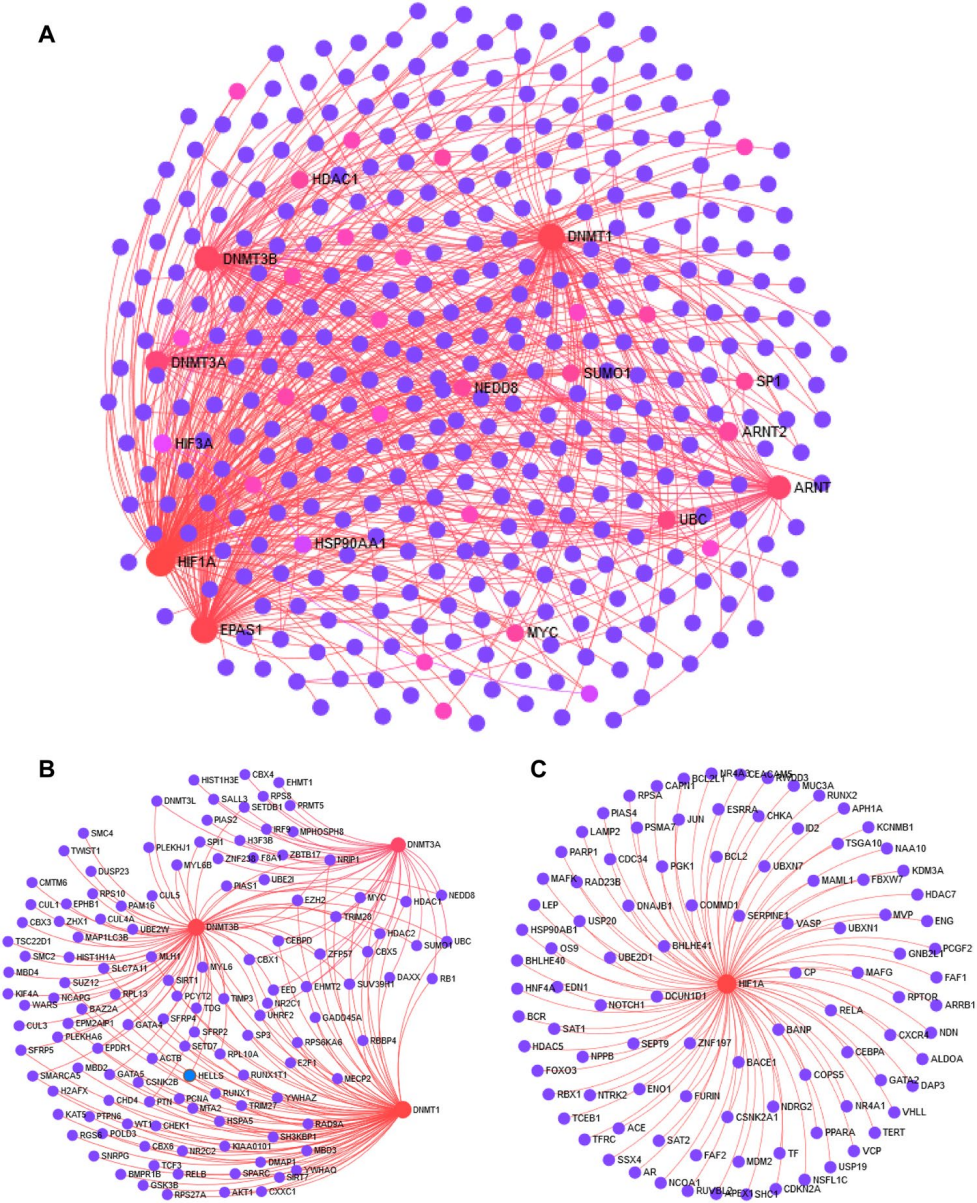
Interacting PPI network analysis of DNMT and HIF family genes. (**A**) Zero-order interaction network of shared DEGs obtained from meta-analysis using force-directed algorithm with minimum overlap layout (**B**) PPI Subnetwork of proteins associated with DNMT family genes (**C**) PPI Subnetwork of proteins associated with HIF1A. The subnetworks were drawn using the module extractor tool of NetworkAnalyst.

PPI network analysis was conducted to understand the relation between the two gene families based on the intermediate genes of the biological network. NetworkAnalyst, a web based tool was implemented to generate a protein-protein interaction (PPI) network by integrating the InnateDB interactome with the original seed of two gene families HIF (HIF1A, EPAS1, HIF3A, ARNT and ARNT2) and DNMT (DNMT1, DNMT3A and DNMT3B). An expanded PPI network was generated with 378 nodes representing the proteins and 525 edges representing the interaction between these proteins, for better visualization of the network “Zero order” interaction network was created (Fig. 5A). As shown in Supplementary Table 4 the most highly ranked nodes (interacting gene) in the interaction network, based on network topology measures, were Ubiquitin C (UBC) with Betweenness centrality = 4984.12; Degree = 6 and Sp1 Transcription Factor (SP1) with Betweenness centrality = 2094.78; Degree = 4 followed by Neural Precursor Cell Expressed, Developmentally Down-Regulated 8 (NEDD8) with Betweenness centrality = 2042.71; Degree = 4 and Small Ubiquitin-Like Modifier 1 (SUMO1) with Betweenness centrality = 2042.71; Degree = 4, Supplementary Table 4 represents the list of top ten hub genes based on network topology scores. Using the module explorer tool, most important modules were extracted as sub networks which included DNMT module (130 nodes and 248 edges) and HIF1A (108 nodes and 234 edges). DNMT and HIF1A sub network/modules are expected to perform independent biological functions by forming protein complexes along with its interacting partner genes in the PPI (Fig. 5B,C).

### Gene set enrichment analysis of the interacting genes in the DNMT-HIF PPI network indicates thrombosis associated pathways

To identify the thrombosis related gene signatures associated with DNMT-HIF PPI network, we briefly conducted GSEA analysis on the complete set of interacting genes from the network using EnrichR tool. In the analysis “Hemostasis (R-HSA-109582)” with overlap (33/552) was observed to be the most significant pathway with several important genes like Serpin Family E Member 1 (SERPINE1); GATA Binding Protein 4 (GATA4); Nitric Oxide Synthase 3 (NOS3); Coagulation Factor XII (F12) and Transferrin (TF). Other important pathways were; “Angiogenesis (P00005)” with overlap (13/142); “NF-kappa B signaling pathway (hsa04064)” with overlap (10/93) and “VEGF, Hypoxia, and Angiogenesis (h_vegfPathway)” with overlap (08/302). Table 4 depicts the thrombosis related pathways and associated genes.

**Table 4:** Top thrombosis related pathway obtained from the enrichment analysis of the genes associated with the interacting PPI of the HIF and DNMT family genes.

GSEA	Pathway/Term ID	Overlap	Associated genes	GSEA library	AdjP-value
**Enriched Thrombosis associated pathways**					
Hemostasis	R-HSA-109582	33/552	APP;HDAC2;SPARC;KDM1A;SHC1;SRC;HDAC1;SERPINE1; GATA5;GATA4;ARRB1;SLC7A11;GATA2;SIN3A;KCNMB1; LAMP2;AKT1;TIMP3;EP300;CREBBP;CBX5;HSPA5;NOS3; F12;YWHAZ;VEGFA;TF;MAFG;KIF4A;PTPN6;MAFK; ALDOA;TP53	Reactome	7.19E-08
Angiogenesis	P00005	13/142	GSK3B;JUN;NOTCH1;SHC1;NOS3;SRC;STAT3;ETS1; VEGFA;APC;AKT1;CTNNB1;EPHB1	Panther	2.52E-05
NF-kappa B signaling pathway	hsa04064	10/93	PIAS4;UBE2I;CSNK2A1;PARP1;CSNK2B;BCL2; IRAK4;RELA;RELB;BCL2L1	KEGG	5.11342E-05
VEGF, Hypoxia, and Angiogenesis	h_vegfPathway	08/302	HSP90AA1;NOS3;SRC;SHC1;ARNT;AKT1;VHL;VEGFA	Biocarta	6.10235E-07
TPO Signaling Pathway	h_TPOPathway	05/42	STAT5A;JUN;CSNK2A1;SHC1;STAT3	Biocarta	0.000280854

List of top thrombosis associated pathways along with its related genes and the GSEA library from which the term was obtained.

## Discussion

The present study revealed miRNAs upstream to hypoxia and methylation by linking miRNA regulation of hypoxia (via HIF gene family members) and methylation (via DNMT gene family members) suggesting the common miRNA signatures followed by the global validation of hypoxia related miRNA signatures using miRNA microarray meta-analysis of hypoxia induced human samples. We further conclude the study by looking into the thrombosis related terms and pathways enriched during protein-protein interaction (PPI) network analysis of these two sets of gene family. The miRNAs are mostly identified on the basis of its complementarity to the 3’UTR sequence of the mRNA under consideration. Other requirements for the miRNA-mRNA binding are based on the free energy between the miRNA-mRNAhybrid, target site accessibility to the miRNA and conservation of the seed sequence throughout the species^18^. Here, we attempt to decipher shared miRNAs through different *in silico* tools like miRDB, Targetscan, RNA22 and Targetminer to identify putative miRNAs. Such algorithms are highly accurate and assist to curtail the targets from thousands to few. The strategy to predict miRNAs-mRNA association has previously been adopted by others for different type of cancers^19,20^. Moreover, the gene expression regulation at the non-coding RNAs level, being a contemporary area of research, has been provided new targets for pre-clinical as well as therapeutic studies. A recent study from our group has indicated that the restoration of miR-145 levels in humans could be considered as a promising therapeutic target for VTE^21^.

The initial analysis in the phase I provided us 30 high confidence miRNAs based on the conservation score being shared between HIF family genes (including HIF1A, EPAS1, HIF3A, ARNT and ARNT2) and DNMT family genes (including DNMT1, DNMT3A and DNMT3B), as shown in Supplementary Table 1. Further, target prediction of these 30 shared miRNAs provided a list of thousands of target genes- a methodology that has been adopted previously by others^22^. Next, the target genes were subjected to functional enrichment analysis to identify genes that are over-represented among a large set of genes. Functional analysis of miRNA target genes revealed regulation via various pathways. Our findings, in agreement to other studies, suggest that the regulation of metabolic process (GO:19222) affects methylation by regulation of one-carbon (methyl) donor production like SAM^23^. Major transcription factor family- HIF organizes regulation of gene expression (GO:10468) and regulate the downstream genes during hypoxia^24^. Further, gene set enrichment analysis (GSEA) of these target genes generated a list of enriched pathways. Ras signaling pathway, together with hypoxia, regulate RECK-a tumor suppressor gene, interestingly via miRNAs^25^. Signaling events mediated by VEGFR1 and VEGFR2 are also associated to hypoxia as the transcription of VEGF upregulates during hypoxia and it is one of the top enriched pathway.

Networks, in biology, are primarily represented by graphs, where several nodes are connected from the inter-nodes, the closeness centrality is the shortest path to the node from internode, while betweenness centrality is the measure of how many times a node serves as the bridge between two components, and together they signify interaction between the two. Identification of hub miRNAs by network analysis is an attempt for better understanding of miRNA functioning during diseases. In this study, we performed network analysis to deduce the most important hub miRNAs shared between hypoxia and methylation based on complex centrality scoring. Here, we did prioritization of the shared miRNAs and mRNA targets via the state-of-the-art prioritization algorithm for hub miRNA selection. One of the study published from our group have applied a part of this analysis strategy for miRNA prediction and prioritization by using network biology, followed by the biological validation of the in silico validation both *in vivo* (in thrombosis induced rats) and human VTE patients^21^. Target gene-miRNA network on cytoscape suggested miR-129, miR-19b and miR-23b as hub miRNAs, based on betweeness and closeness centrality. miR-129-5p is linked with hypoxia, when HL1 cells and human cardiomyocytes showed dowenregulated miR-129-5p during oxidative stress. Both microR-129-5p and miR-19b have been identified as a biomarker for heart failure and diabetic cardiomyopathy respectively, as suggested by the literature and this was further validated by inducting hypoxia to their respective cell culture models^26,27^. Similarly, hypoxia gradually increases miR-23b in cardiomyocyte and prevented the growth by apoptosis^28^. Among the two gene families, DNMT3A and HIF1A showed maximum cardiovascular diseases related targets in Table 1, searched via TargetScan database. Further, the FASTA sequences of these two genes were subjected to RNAhybrid (version: 2.2), a tool previously cited by others for functional analysis of miRNA targets^29^. Based on the minimum free energy, the most stable interaction was found to be between- DNMT3A-miR129; DNMT3A-miR19b; HIF1A-miR19b and HIF1A-miR330. Individually the targets and their respective miRNAs have been studied previously, however, together the role of these genes in thrombosis has to be studied.

Besides, in this study we attempted to emphasize the role of hypoxia and methylation, per se, in accordance to thrombosis. We have reports supporting our study hypothesis which maintain that on induction to high altitude/ hypoxic exposure, there is alteration in the blood hemostasis^30–32^. Hypoxia is responsible for hypercoagulable state which may result in increased propensity of thrombosis^33–35^. As evident from the previous publication of our group, hypoxia induces altered platelet proteome/reactivity, which correlates with a prothrombotic phenotype. In this article the author have dissected the molecular mechanism behind hypoxia-induced platelet aggregation and activation of blood coagulation^36^. Similarly, another article, recently published from our lab, conducted gene expression microarray profiles in DVT patients, who developed the disease either at sea level (SL-DVT) or at high altitude (HA-DVT) locations. Comparison of the expression profile in the two sets of disease groups revealed that the differential expression of hypoxia-responsive genes in HA-DVT could be a determining factor to understand the pathophysiology of DVT at high altitude^3^. Aberrant miRNA expression during different environmental stress has been studied previously, since, the trigger selected was hypoxia; we followed the studies with altered miRNA expression signatures during hypoxic conditions. In order to assess the shared miRNAs among hypoxia induced samples, a miR-microarray meta-analysis (phase II) was conducted as a part of the global validation of the HIF-miRNA axis part of phase I. Microarray meta-analysis is based on powerful statistical methods to generate a list of common genes or miRNAs in miR-meta-analysis, to identify an intersecting crossroad between multiple datasets. miRNA-microarray datasets were retrieved from publicly available online databases to check the expression profiles of miRNAs during hypoxia and to observe its concomitant effect, a methodology adopted by others as well^37^. Besides there are an array of published research article, where miRNA meta-analysis of microarray data have been conducted to find the most important regulatory miRNAs differentially expressed in case of several diseases and conditions, some of the examples are in hepatocellular carcinoma^38,39^, colorectal cancer^40^, miRNA dysregulation in epilepsy^41^ and prostate cancer^22^ among others. Our results showed 29 differentially expressed miRNAs, including 20 upregulated and 9 downregulated miRNAs across the three datasets shown in Table 3. Interestingly, the top overexpressed miRNAs- miRNA-210, miRNA-483 and miRNA-361 were found to be associated with cardiovascular disease risks and have been reported in various cardiovascular diseases^42–44^. Subsequently, we performed enrichment analysis with the help of miRNA Enrichment Analysis and Annotation (miEAA) tool. This tool has been previously utilized for clustering and analyzing the miRNAs for potential pathways involved in acquired cardiomyopathy^45^. The tool scrutinizes the list of over and underexpressed miRNAs from the published datasets and observes potential association between diseases and pathways. The “integrin mediated cell adhesion” is a target for the treatment of thrombosis. The integrins mediate platelet adhesion via binding to adhesive proteins followed by platelet aggregation^46,47^. “Dilated cardiomyopathy” has earlier been linked with miR-208, miR-548-c, miR-30-c and was the most highly enriched disease term observed in the miR meta-analysis^48–50^. However, in our results, hsa-miR-484, hsa-miR-125a-3p and hsa-miR-28-5p were found to be associated with dilated cardiomyopathy.

The PPI network data is a mathematical representation of the interactions between two or more proteins-irrespective of their families and enable us to detect the proteins and pathways having an impact on the downstream signaling. PPIs have facilitated us to look into the connections between proteins from a group of proteins. A PPI network was generated between two gene families viz. HIFs and DNMTs. As shown in Fig. 5A, a zero order interaction network is generated where the seed gene list was generated from the DEGs acquired from the microarray datasets analysis. Next, a PPI subnetwork of both DNMT and HIF1A was constructed, here both DNMT and HIF1A was the ‘continent’ while the interacting proteins were ‘islands’. All the analysis was further conducted on the continent by module extractor tool of NetworkAnalyst. The top proteins in the PPI were Ubiquitin C-which is extensively studied in platelets responsiveness during stress and Sp1transcription factors^51^. Interestingly, megakaryocyte specific knockout of Sp1/Sp3 transcription factors display thrombocytopenia and platelet dysfunction in mice^52^.

PPI network analysis (phase III) was performed to establish missing link between the role of methylation and hypoxia all together in thrombosis. Therefore, in this phase of our study we conducted PPI network analysis of DNMT and HIF gene family, revealing interacting intermediate genes network. Gene set enrichment analysis was done using EnrichR tool using the interacting genes in the DNMT-HIF PPI network as seed. Interestingly, the results showed the most significant pathway as “Hemostasis (R-HSA-109582)”. Hemostasis is a steady state, where blood maintains its fluidity in normal physiological conditions. It is widely accepted that changes in the hemostasis accelerates coagulation related disorders, thrombosis being one. Hemostasis and thrombosis go hand in hand as per many studies^53^. Angiogenesis, the next most significant pathway, has been linked with thrombosis during cancer^54^.Target activation of NF-kappa B subunits in NF-kappa B signaling pathway has a protective impact on athero-thrombogenesis^55^. VEGF, Hypoxia, and Angiogenesis pathway, on the other hand, is involved in interaction between cancer and thrombosis^56^. Next, the TPO Signaling Pathway is associated with megakaryopoiesis- a process involving hematopoietic stem cells to generate megakaryocyte followed by the production of platelets^57^.

Any study would have its limitations, so does ours and it is important to highlight them. The heterogeneity in the datasets of microarray is one of the major limitations followed by the confounding factors associated with the sample type difference, which may have skewed the analysis. As mentioned in the method section, the three datasets used for miRNA microarray meta-analysis were using different types of human cells viz. GSE47532 (breast cancer cell line MCF-7), GSE60432 (human placental trophoblast cells) and GSE68593 (Hepatocellular Carcinoma cells). Since, there is no robust method or tool available to adjust for sample type difference, these effects are hard to assess due to the paucity of available datasets in human. Although individual normalization for each dataset was conducted along with the batch effect correction, however, we cannot deny the confounding factors in the study. Statistically individual gene expression profiling studies are limited by both biological (e.g., use of gene expression profiles in one cell/tissue) and technical (e.g., use of single expression analysis platform) biases, leading to inconsistent results among studies and hindering the broad application of their findings and translation into clinical practice. Moreover, microarray data integration continues to be a challenging problem because of the inherent heterogeneity of individual datasets, which we tried to resolve in our study by individual data preprocessing and normalization followed by batch effect correction before proceeding to meta-analysis. Although many sophisticated algorithms have been published in recent years, no single statistical method is optimal. However, the major advantage of microarray data integration is increased statistical power for detecting DE miRNAs, while also providing more robust, reproducible and accurate predictions^58^. Secondly, the lack of global validation of the methylation-miRNA axis via comprehensive miRNA microarray meta-analysis due to paucity of human microarray datasets remains another limitation of the current study. In conclusion, this study is the first attempt to our knowledge that links miRNA regulation of hypoxia (via HIF gene family members) and methylation (via DNMT gene family members) implicating the common miRNA signatures. Moreover, this study also incorporates the global validation of the hypoxia related miRNA signatures using miRNA microarray meta-analysis of the hypoxia induced human samples versus healthy samples. We further conclude the study by looking into the thrombosis related terms and pathways enriched during protein-protein interaction network analysis of these two sets of gene. Interestingly, the study has generated a novel database of candidate miRNA signatures shared between hypoxia and methylation; and their (DNMT & HIF family genes) relation to thrombotic pathways, which might aid in the development of potential therapeutic biomarkers.

## Methods

The study involves three phase analysis linking shared signatures of miRNA between hypoxia and methylation, intrinsic biological validation of the hypoxia-miRNA axis and finally relating its association with thrombotic terms/pathways. The phases of this in silico study are highlighted as follows;

Phase I- Shared miRNA analysis between HIF and DNMT gene family: In this phase we obtained that HIF and DNMT gene family is regulated via a pool of shared miRNAs. This was followed by target prediction of the shared miRNA and their enrichment analysis. Furthermore, network prioritization of these shared miRNAs was conducted to reveal the most important hub miRNAs. Candidate hub miRNA-target gene secondary structure analysis of binding with DNMT and HIF family genes was also conducted to show the most stable interacting miRNA-DNMT/HIF gene pairs. Figure 1A outlines the analysis strategy for phase I.

Phase II- miRNA microarray meta-analysis of the hypoxia induced human samples (Validation of findings from phase I): This phase takes care of the global validation of the findings from phase I; specifically the hypoxia-miRNA axis. Here, we would like to clarify that we looked to validate the methylation-miRNA axis via similar approach, however, the lack of human microarray datasets evaded us to validate this axis (methylation-miRNA). Therefore, in this phase comprehensive gene expression meta-analysis of miRNA microarray datasets of hypoxia samples reveals differentially expressed miRNAs, which were also present as putative miRNAs targeting HIF family gene members. The DE miRNAs obtained were then subjected to enrichment analysis using miEAA tool. Supplementary Table S5 is the link between phase I and phase II. Figure 1B outlines the analysis strategy for phase II.

Phase III- PPI network analysis associates HIF and DNMT gene family to thrombosis: PPI network analysis of DNMT and HIF gene family reveals interacting intermediate genes network. Gene set enrichment analysis of the interacting genes in the DNMT-HIF PPI network indicates thrombosis associated pathways. Figure 1C outlines the analysis strategy for phase III.

### miRNA prediction and identification of shared miRNAs between HIF and DNMT gene family

For identification of shared miRNA signatures between hypoxia and methylation, putative miRNA prediction was done for genes associated with Hypoxia-inducible factors (HIFs) family genes (including HIF1A, EPAS1, HIF3A, ARNT and ARNT2) and DNA methyltransferase (DNMT) family genes (including DNMT1, DNMT3A and DNMT3B). Identification of putative miRNA, targeting these gene families was done using miRDB^59^, Targetscan^60^, RNA22^61^ and Targetminer^62^ to select miRNAs with high confidence level, based on its interaction shown in at least three databases and the conservation score across the mammals. For convenience, we considered target gene miRNA interaction which was conserved and defined by its conserved branch length. A curated list of these highly conserved miRNAs targeting both the gene families was prepared and compared across to generate a list of miRNAs being shared among them for further downstream analysis (Supplementary Table 1 and Supplementary Sheet 1). The overall pipeline of the study is outlined in Fig. 1A.

### Shared miRNA target prediction and its gene set enrichment analysis

The list of shared miRNA obtained, was subjected to miRNA target prediction using five different programs, in the present study to ensure high specificity in target prediction. Target prediction was carried out using miRWalk^63^, miRDB, Targetscan, RNA22 and Targetminer. Targets predicted by 4 or 5 out of 5 programs were retained to ensure that only highly validated targets were retained. The programs were chosen to represent different approaches for miRNA target prediction. The miRWalk prediction software identified potential binding sites by looking for possible binding site interaction information (including “central pairing sites”) between genes (encompassing the complete sequence as well as mitochondrial genomes) and miRNAs resulting from the miRWalk algorithm by walking with a heptamer (7nts) seed of miRNA from positions 1 to 6. The program TargetScan 5.1 combines thermodynamics-based modeling of RNA–RNA duplex interactions with comparative sequence analysis to predict miRNA targets conserved across multiple mammalian genomes. TargetScan mainly depends on perfect complementarity to the seed region of miRNA and then extends to complementarity outside the region. The algorithm miRDB uses a newly developed SVM classifier. Upon target prediction, there was a huge list of target genes predicted to bind with the shared miRNAs. Therefore, we used an approach to sort the number of target genes based on the perfectly matched gene-miRNA pair with “seed” score 1, we excluded pairs with seed score 0. We further removed the duplicates among the pairs to generate a final list of miRNA-target genes. The predicted target genes above were submitted to BiNGO^64^ a gene ontology tool which works in the cytoscape environment. BiNGO analysis was conducted to visualize which Gene Ontology (GO) biological process terms are significantly enriched among the set of target genes employing hyper-geometric test statistics. Since it involves multiple testing during enrichment analysis, therefore,false discovery rate (FDR) correction was done by Benjamini and Hochberg method (Fig. 2) and (Supplementary Sheet 2). Lower the P-value, the more significant the correlation; the recommended adjusted P-value cut-off is 0.05. Besides, Gene Set Enrichment Analysis (GSEA) of these target genes was conducted using EnrichR platform (Supplementary Table 2) to identify the most significantly enriched associated terms.

### Hub miRNA analysis using network biology of miRNA-mRNA network

For prioritization of candidate miRNAs shared between the two gene families, the miRNA-target gene network was constructed based on the putative interaction analysis. Only those miRNA-target gene showing perfect match, based on the seed score and have validated interaction based on miRTArbase were selected to construct the network using the Cytoscape 3.4.0 program^65^. The degree was defined as the number of directly linked neighbors. Network analysis of the co-expression network was done using network analyzer^66^ and centiscape^67^ tool on cytoscape. There are three methods employed to create and analyze the network, which includes; the query of list of gene of interest to the associated protein databases such as cPath, second method is to build network by text mining with the help of literature search plugins and the third method is to import a network file, such as a Simple Interaction File (SIF or .sif), Graph Markup Language (GML or .gml), Delimited Text Table, Excel Workbook (.xls) file etc. Since, file import is the most appropriate method for users who want to focus their analysis on network data identified in advance we used this method for network analysis. For creating interaction network, we generated an Excel Workbook (.xls) where we arranged the list of miRNAs (source node) and their target mRNAs (target nodes). We further import this network data into Cytoscape. Using the in-built plugin of cytoscape “networkanalyzer” we created undirected networks. In undirected network there is no direction of the edges. The edges are considered as bi-directional and the classic centralities definition are applied. The correlation (Y) between degree and number of nodes of the network was 0.953 while the R2 value was 0.742. We further analyzed various network parameters of the miRNA-mRNA network which includes; network diameter, network radius, centralization, No. of neighbours, Nodes, network density, heterogeneity and clustering coefficient etc (Supplementary Fig. 2). A list of top ten prioritized miRNAs were generated based on the simple and complex network parameters including degree^68^, betweenness centrality^69^ and closeness centrality^70^ (Fig. 3A,B).

### Secondary structure analysis of candidate hub miRNA-target gene pair

Using the prioritized list of candidate hub miRNA-target gene pair, we selected miR-129-DNMT3A, miR-19b-DNMT3A, miR-19b-HIF1A and miR-330-HIF1A for secondary structure analysis. Thermodynamic principles govern all reactions in biological systems. Therefore, the measurement of minimum free energy makes it possible to assess how strong the binding is between the miRNA and its target mRNA. Thus, the lower the free energy, the greater the RNA:RNA binding, increasing the likelihood that this interaction will actually occur. The free energy is expressed in negative real value and its unit is kcal/mol. This estimation is performed considering that the mRNA adopts a secondary structure, so the site to which a particular miRNA will join must be accessible and stable. Moreover, RNAhybrid was used to calculate the minimum free energy (MFE) of the duplex miRNA:mRNA. RNAhybrid was optimized to show the hybridization at the 3-UTR of the target genes. Illustrations of the miRNA-gene interactions are shown in Table 1. Predictions of miRNA duplex secondary structures were obtained from the RNAhybrid Web service^71^. We also used microRNA.org^72^ for calculating mirSVR to compute a score that represents the effect, a miRNA may have, on expression of target genes.

### MicroRNA datasets and individual data analysis of differentially expressed hypoxia related miRNAs

To analyze the differentially expressed miRNAs in hypoxic condition in humans, we worked on the publically available microarray datasets. In this study, a total of three microRNA datasets related to hypoxic exposure in human samples; GSE47532^73^, GSE60432^74^ and GSE68593^75^ were selected for microarray meta-analysis. In the GSE47532, a total of 3 normoxic control and 8 hypoxia exposed replicates, culture of breast cancer cell line MCF-7, was used to establish the relationship between microRNA expression and HIF binding sites, pri-miRNA transcription and microRNA processing gene expression. microRNA sequencing data and gene expression microarray data were generated from MCF-7 cells submitted to a hypoxia time course (16 h, 32 h and 48 h at 1% Oxygen). In GSE60432, total RNA from 16 normoxic control and 16 hypoxia incubated culture of human placental trophoblast cells were extracted for expression profiling by array. Human placental trophoblasts were dispersed using a trypsin-deoxyribonuclease-dispase/Percoll method, plated in 6-well plates and maintained in standard culture conditions (O_2_ = 20%). After 4 h (defined as time 0) the plates were divided to eitherin continued standard culture conditions, or to culture in hypoxia (O_2_ = 0%). Cells were then harvested at 12 h, 24 h, 48 h and 72 h, and processed for miRNA arrays. For meta-analysis, we divided GSE60432 into 4 individual datasets with hypoxic exposure (12 h, 24 h, 48 h and 72 h at 0% Oxygen). GSE68593 was designed for detection of hypoxia inducible/reducible miRNAs in Hepatocellular Carcinoma cells using Non-coding RNA profiling by array. Total RNA was isolated from HCC cells exposed to hypoxic conditions for 0hrs and 8 h. Both groups contained 3 independent replicates. The detailed information on the datasets used in this study is summarized in Table 2. The microRNA microarray datasets were obtained from GEO NCBI. The three datasets used for miRNA microarray meta-analysis used different types of human cells viz. GSE47532 (breast cancer cell line MCF-7), GSE60432 (human placental trophoblast cells) and GSE68593 (Hepatocellular Carcinoma cells). Although individual normalization for each dataset was conducted along with the batch effect correction, however, we cannot deny the confounding factors in the study. Well established ComBat procedure^76^ was employed on pre-processed and normalized datasets to reduce study-specific batch effect across the biological groups in selected datasets for analysis. Supplementary Fig. 1A,B depicts the PCA before and after batch effect correction.

### Comprehensive meta-analysis of hypoxia related miRNA microarray datasets

Using the three datasets related to hypoxia exposed miRNA expression, a comprehensive microarray meta-analysis was conducted using Integrative Meta-Analysis of Expression Data (INMEX), a web interface for integrative meta-analysis^77^. It is worthwhile mentioning here that INMEX tool does not have the functionality to convert miRNA probes therefore; we selected only those datasets with common platform and probe name version. After matching all probes, quantile normalization followed by log2 transformation was done with individual datasets as the part of preprocessing, To ensure that there was equal distribution among the individual datasets, we visualized box plots and Principal Component analysis (PCA) plots (Supplementry Fig. 1). Firstly, DE analysis was conducted on individual datasets using the moderated t test based on the Limma algorithm^78^ using a false discovery rate of 0.05, a significance of P < 0.05 as the selection criteria for significant DE miRNAs. In INMEX, the results from individual microarray dataset analyses are only for reference comparison and not required for meta-analysis in the subsequent steps. After the normalization, preprocessing and data integrity check of the individual datasets, we conducted differential expression meta-analysis across hypoxia exposed and control samples combining p-value (Fisher’s method). This method of gene expression meta-analysis is considered to be the most popular, since, it generates most consistent biological results by considering both the direction and magnitude of gene expression. Calculating and combining P-values from multiple studies as a means of data integration has long been used in the meta-analysis of microarray data^79^. This approach is simple to calculate and flexible to use. Fisher’s method can be easily interpreted, whereby larger scores reflect greater differential expression. “Pattern extractor” tool from INMEX was used to visualize the subset of DE miRNAs as heatmap, besides; we also generated venn diagram and chord diagram using the visualization tool for multiple datasets for better visualization of our findings (Fig. 4). Figure 1B shows the overall pipeline used in miRNA microarray meta-analysis of hypoxia datasets.

### Enrichment analysis of differentially expressed miRNAs using the miRNA Enrichment Analysis and Annotation tool (miEAA)

Enrichment analysis of biological pathways associated with differentially expressed miRNAs, obtained by meta-analysis of publically available gene expression datasets, was performed using the miEAA tool (https://ccb-compute2.cs.uni-saarland.de/mieaa_tool/)^80^. miRNA Enrichment Analysis and Annotation tool (miEAA) is a web-based application, that offers a variety of commonly applied statistical tests such as over-representation analysis and miRNA set enrichment analysis, which is similar to Gene Set Enrichment Analysis. Besides the different statistical tests, miEAA also provides rich functionality in terms of miRNA categories. To further investigate the function of the DE miRNAs, they were also mapped to the most significant disease enrichment pathway which is based on the role of inputs miRNAs available from pubmed (Supplementary Table 3).

### Interacting PPI network analysis of DNMT and HIF family genes

We constructed a protein-protein interaction network between DNMT and HIF family genes using NetworkAnalyst^81^. This analysis was done to find out the intermediate interacting proteins associated with the two gene families. This system biology based network biology also generated a list of most important hub genes by taking the advantage of common functions for network topology (viz. Degree and betweenness centrality) and module analyses approaches. Briefly, the complete list of genes of both DNMT and HIF family were uploaded into the web- based server of NetworkAnalyst and zero order interactors were considered in the network as the PPI generated from the original seed (DNMT1, DNMT3A and DNMT3B, HIF1A, EPAS1, HIF3A, ARNT and ARNT2) was having greater than 2000 node and edge interactions. The network of this size creates “hairball effect” and doesn’t allow proper visualization of interaction network. Network centrality topological measures— which include degree and betweenness centrality are the features of NetworkAnalyst that help in identification of highly interconnected hub nodes. The nodes (genes) with greater centrality score is supposed to control most of the information flowing in the network, representing the critical points of the network^82^. Using “module explorer” we extracted the top two sub networks from the parent network of PPI between HIF and DNMT family genes (Fig. 5). Figure 1C demonstrate the overall steps performed for this phase of analysis.

### GSEA analysis of the interacting protein in the PPI to find the thrombosis related terms

We further created a list of interacting proteins from the PPI generated between HIF and DNMT family genes. These protein/genes were used to discern their implication on thrombosis related terms. Exploiting the extended gene set library of EnrichR platform^83^ we performed the functional enrichment analysis. EnrichR web-tool integrates several databases for functional analysis of the set of genes in question; it includes Gene ontology^84^ and various pathway analysis libraries like Kyoto Encyclopedia of Genes and Genomes pathway (KEGG), Reactome pathway, Wikipathway, panther and biocarta. The top enriched terms associated with thrombosis along with the interacting genes from PPI is listed in Table 4. Enriched annotations/pathways were selected/ranked based upon combined score which was calculated by the EnrichR platform following Z-score permutation background correction on the Fischer Exact Test p-value.

### Statistical analyses

The meta-analysis was performed using the web-based tool—INMEX. Differential expression meta-analysis across hypoxia exposed and control samples was done by combining p-value (Fisher’s method). An adjusted p value of 0.05, based on the false discovery rate using the Benjamini–Hochberg procedure was used to select DE miRNAs. Selected statistical test for multiple testing during functional enrichment analysis: Hypergeometric test (right-sided) and selected correction: Bonferroni/ Benjamini& Hochberg False Discovery Rate (FDR) correction. Significantly enriched pathways were identified using hypergeometric tests and an adjusted p-value ≤ 0.05 was applied as the cut-off value for statistical significance.

## Supplementary information

Supplementary Information

Dataset 1

Dataset 2

Dataset 3
