# Supplementary material for: micro-RNAs dependent regulation of DNMT and HIF1α gene expression in thrombotic disorders
Source: Sci Rep. 2019 Mar 20;9:4815. doi: 10.1038/s41598-018-38057-6 (PMC6426883; doi:10.1038/s41598-018-38057-6)
Supplement: Supplementary file 1 — Supplementary Information [file 41598_2018_38057_MOESM1_ESM.docx]

**Supplementary information**

**micro-RNAs dependent regulation of DNMT and HIF1a gene expression in thrombotic disorders**

Aatira Vijay^1#^, Prabhash Kumar Jha^1#^, Iti Garg^1^,Manish Sharma^1^, Mohammad Zahid Ashraf^1, 2^and Bhuvnesh Kumar^1*^

1. Defence Institute of Physiology and Allied Sciences, DRDO, Delhi, INDIA
2. Department of Biotechnology, JamiaMilliaIslamia, Delhi, INDIA

*Address for Correspondence:

Bhuvnesh Kumar, Defence Institute of Physiology & Allied Sciences, DRDO

Lucknow Road, Timarpur, Delhi, INDIA-110054

E-mail: bhuvnesh.drdo@gmail.com

#Authors contributed equally to the work.


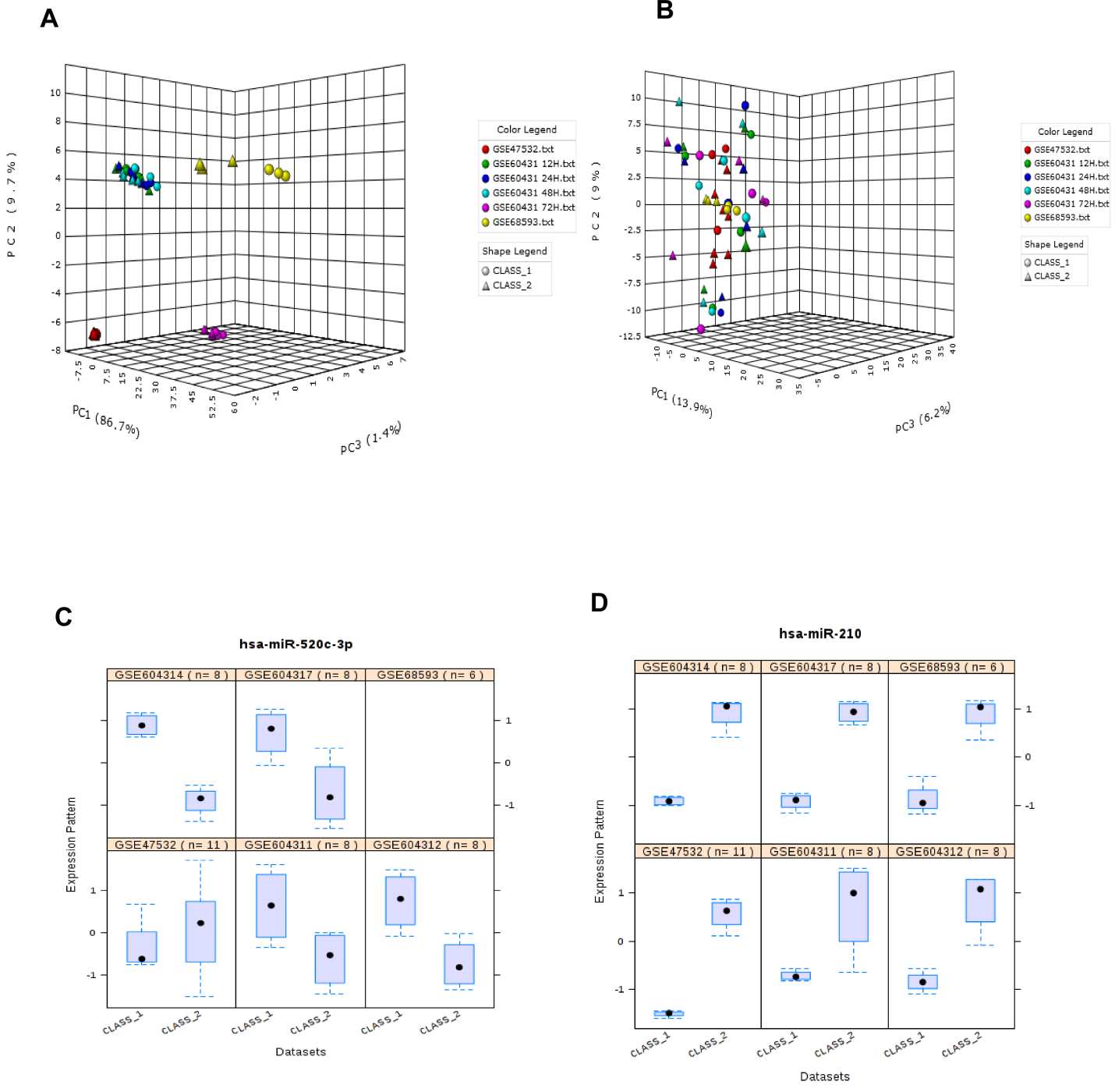


**Supplementary Figure 1- Batch effect analysis**- Illustration of PCA plots as validation tools for batch effect removal. Plot of principal components: (a) before batch effect removal and (b) after batch effect removal (using Combat method). (c) Showing the expression of top upregulated miRNA in individual datasets and (d) Showing the expression of top downregulated miRNA in individual datasets.

**Supplementary Figure 2**- The power law frequency distribution of the network was obtained with the “Network Analysis” tool of Cytoscape showing simple topological parameters.

| DNMT | | | | | | HIF | | | | |
| --- | --- | --- | --- | --- | --- | --- | --- | --- | --- | --- |
| Shared miRNA | **Position in the UTR** | **seed match** | **context++ score** | **conserved branch length** | **Pct** | **Position in the UTR** | **seed match** | **context++ score** | **conserved branch length** | **Pct** |
| hsa-miR-29c-3p | 1202-1209 | 8mer | -0.42 | 3.397 | 0.75 | 857-863 | 7mer-m8 | -0.25 | 4.707 | 0.85 |
| hsa-miR-29b-3p | 862-868 | 7mer-m8 | -0.33 | 6.65 | 0.87 | 857-863 | 7mer-m8 | -0.25 | 4.707 | 0.85 |
| hsa-miR-29a-3p | 1202-1209 | 8mer | -0.42 | 3.397 | 0.75 | 857-863 | 7mer-m8 | -0.25 | 4.707 | 0.85 |
| hsa-miR-148b-3p | 48-55 | 8mer | -0.42 | 2.935 | 0.44 | 1146-1153 | 8mer | -0.2 | 3.358 | 0.63 |
| hsa-miR-152-3p | 48-55 | 8mer | -0.42 | 2.935 | 0.44 | 1146-1153 | 8mer | -0.2 | 3.358 | 0.63 |
| hsa-miR-148a-3p | 48-55 | 8mer | -0.42 | 2.935 | 0.44 | 1146-1153 | 8mer | -0.2 | 3.358 | 0.63 |
| hsa-miR-142-5p | 263-269 | 7mer-m8 | -0.11 | 3.161 | 0.15 | 1982-1989 | 8mer | -0.17 | 3.979 | 0.28 |
| hsa-miR-5590-3p | 263-269 | 7mer-m8 | -0.11 | 3.161 | 0.15 | 171-177 | 7mer-m8 | -0.02 | 3.502 | 0.2 |
| hsa-miR-495-3p | 322-328 | 7mer-m8 | -0.02 | 3.086 | N/A | 1284-1290 | 7mer-1A | -0.01 | 6.339 | N/A |
| hsa-miR-5688 | 322-328 | 7mer-m8 | -0.02 | 3.086 | N/A | 1284-1290 | 7mer-1A | -0.01 | 6.339 | N/A |
| hsa-miR-129-5p | 373-379 | 7mer-m8 | -0.02 | 3.337 | <0.1 | 1869-1875 | 7mer-m8 | -0.03 | 3.671 | < 0.1 |
| hsa-miR-194-5p | 768-774 | 7mer-m8 | -0.12 | 5.572 | 0.59 | 1656-1662 | 7mer-1A | -0.01 | 0.572 | < 0.1 |
| hsa-miR-199b-3p | 837-843 | 7mer-m8 | -0.28 | 6.344 | 0.85 | 1949-1955 | 7mer-1A | -0.15 | 3.604 | 0.51 |
| hsa-miR-199a-3p | 837-843 | 7mer-m8 | -0.28 | 6.344 | 0.85 | 1949-1955 | 7mer-1A | -0.15 | 3.604 | 0.51 |
| hsa-miR-3129-5p | 837-843 | 7mer-m8 | -0.2 | 6.344 | 0.85 | 1949-1955 | 7mer-1A | -0.15 | 3.604 | 0.51 |
| hsa-miR-493-5p | 842-848 | 7mer-1A | -0.01 | 3.805 | N/A | 1225-1231 | 7mer-1A | -0.06 | 4.653 | N/A |
| hsa-miR-130a-5p | 1398-1404 | 7mer-m8 | -0.02 | 2.913 | 0.17 | 580-587 | 8mer | -0.1 | 4.203 | 0.49 |
| hsa-miR-23b-3p | 1398-1404 | 7mer-m8 | -0.02 | 2.913 | 0.17 | 580-587 | 8mer | -0.11 | 4.203 | 0.49 |
| hsa-miR-23a-3p | 1398-1404 | 7mer-m8 | -0.02 | 2.913 | 0.17 | 580-587 | 8mer | -0.11 | 4.203 | 0.49 |
| hsa-miR-23c | 1398-1404 | 7mer-m8 | -0.02 | 2.913 | 0.17 | 580-587 | 8mer | -0.11 | 4.203 | 0.49 |
| hsa-miR-340-5p | 2024-2030 | 7mer-1A | -0.01 | 4.39 | N/A | 663-669 | 7mer-1A | -0.01 | 6.339 | N/A |
| hsa-miR-138-5p | 3056-3062 | 7mer-m8 | -0.02 | 3.303 | 0.42 | 632-638 | 7mer-1A | -0.28 | 9.27 | 0.89 |
| hsa-miR-19a-3p | 5479-5485 | 7mer-1A | -0.05 | 3.988 | 0.58 | 606-612 | 7mer-1A | -0.2 | 5.255 | 0.58 |
| hsa-miR-19b-3p | 5479-5485 | 7mer-1A | -0.05 | 3.988 | 0.58 | 606-612 | 7mer-1A | -0.2 | 5.255 | 0.58 |
| hsa-miR-26b-5p | 5709-5716 | 8mer | -0.16 | 7.028 | 0.94 | 1772-1778 | 7mer-1A | -0.12 | 4.149 | 0.59 |
| hsa-miR-1297 | 5709-5716 | 8mer | -0.16 | 7.028 | 0.94 | 1772-1778 | 7mer-1A | -0.12 | 4.149 | 0.59 |
| hsa-miR-26a-5p | 5709-5716 | 8mer | -0.16 | 7.028 | 0.94 | 1772-1778 | 7mer-1A | -0.12 | 4.149 | 0.59 |
| hsa-miR-4465 | 5709-5716 | 8mer | -0.16 | 7.028 | 0.94 | 1772-1778 | 7mer-1A | -0.12 | 4.149 | 0.59 |
| hsa-miR-330-3p | 5859-5865 | 7mer-1A | -0.01 | 3.728 | N/A | 576-582 | 7mer-m8 | -0.02 | 3.868 | N/A |
| hsa-miR-323a-3p | 6108-6114 | 7mer-1A | -0.01 | 3.807 | N/A | 4180-4186 | 7mer-1A | -0.01 | 4.848 | N/A |

**Supplementary Table 1- Shared miRNA between HIF and DNMT family genes.** A list of shared miRNAs and its interaction details including; position in UTR, seed match and conserved branch length.

| Enrichment Term | Pathway/Term ID | Overlap | GSEA library | AdjP-value |
| --- | --- | --- | --- | --- |
| Enriched Pathways | | | | |
| Ras signaling pathway | hsa04014 | 136/227 | KEGG | 2.22E-07 |
| Neurotrophin signaling pathway | hsa04722 | 78/120 | KEGG | 1.66E-06 |
| Signaling events mediated by VEGFR1 and VEGFR2 | Homo sapiens | 45/68 | NCI-NATURE | 0.000685 |
| EGF receptor signaling pathway | P00018 | 72/109 | Panther | 3.23E-06 |
| Endothelin signaling pathway | P00019 | 48/75 | Panther | 0.000474 |
| Developmental Biology | R-HSA-1266738 | 411/786 | Reactome | 8.15E-10 |
| Axon guidance | R-HSA-422475 | 283/515 | Reactome | 1.54E-09 |
| NFAT and Hypertrophy of the heart | h_nfatPathway | 28/44 | Biocarta | 0.04853 |
| DNA Damage Response (only ATM dependent) | WP710 | 72/111 | WikiPathway | 1.91E-05 |
| Insulin Signaling | WP481 | 95/160 | WikiPathway | 5.07E-05 |

**Supplementary Table 2- Top enriched terms and biological pathways identified by functional analysis of the target genes associated with the shared miRNAs.** Overlap: indicates the number of hits from the meta-analysis compared to each curated gene set library. Gene set functional analysis was performed using extended libraries of the EnrichR tool. Enriched terms and pathways were ranked based on the adjusted p-value. KEGG: Kyoto Encyclopedia of Genes and Genomes; GO: gene ontology biological process; GSEA: Gene Set Enrichment Analysis.

| **GSEA** | **Pathway/Term ID** | **AssociatedmiRNAs** | **GSEA library** | **AdjP-value** |
| --- | --- | --- | --- | --- |
| **Enriched Terms** | | | | |
| Integrin mediated cell adhesion | WP185 | hsa-miR-125a-5p; hsa-miR-140-5p; hsa-miR-484; hsa-miR-34c-5p; hsa-miR-518b; hsa-miR-28-5p | miRWalk | 0.0106009 |
| Neurotrophin signaling pathway | hsa04722 | hsa-miR-342-3p; hsa-miR-125a-5p; hsa-miR-140-5p; hsa-miR-107; hsa-miR-193a-5p; hsa-miR-484; hsa-miR-34c-5p; hsa-miR-518b; hsa-miR-28-5p | miRWalk | 0.00785022 |
| MAPK signaling pathway | WP382 | hsa-miR-342-3p; hsa-miR-125a-5p; hsa-miR-140-5p; hsa-miR-107; hsa-miR-484; hsa-miR-34c-5p; hsa-miR-518b; hsa-miR-28-5p | miRWalk | 0.0214545 |
| lipid particle | GO:0005811 | hsa-miR-342-3p; hsa-miR-125a-5p; hsa-miR-140-5p; hsa-miR-107; hsa-miR-193a-5p; hsa-miR-484; hsa-miR-34c-5p; hsa-miR-518b; hsa-miR-28-5p | GO | 0.00294724 |
| regulation of cell differentiation | GO:0045595 | hsa-miR-107; hsa-miR-484; hsa-miR-34c-5p; hsa-miR-28-5p | GO | 0.00884173 |
| **Associated enriched diseases** | | | | |
| dilated cardiomyopathy | - | hsa-miR-484; hsa-miR-125a-3p; hsa-miR-28-5p | Pubmed | 0.0153257 |
| colon cancer | - | hsa-miR-483-3p; hsa-miR-342-3p; hsa-miR-520d-3p; hsa-miR-107; hsa-miR-193a-5p; hsa-miR-484; hsa-miR-516a-5p; hsa-miR-34c-5p; hsa-miR-125a-3p; hsa-miR-28-5p | Pubmed | 0.0121253 |
| glioma | - | hsa-miR-582-5p; hsa-miR-193a-5p; hsa-miR-516a-5p; hsa-miR-518b | Pubmed | 0.014652 |

**Supplementary Table 3- Enrichment analysis of differentially expressed miRNAs using the miRNA Enrichment Analysis and Annotation tool (miEAA).** List of top pathways associated pathways along with its related miRNAs and the GSEA library from which the term was obtained.

| Label | Degree | Betweenness |
| --- | --- | --- |
| UBC | 6 | 4984.12 |
| SP1 | 4 | 2094.78 |
| NEDD8 | 4 | 2042.71 |
| SUMO1 | 4 | 2042.71 |
| MYC | 4 | 1642.49 |
| HDAC1 | 4 | 1642.49 |
| HSP90AA1 | 4 | 16.75 |
| HSPA4 | 3 | 1518.64 |
| SUMO2 | 3 | 1518.64 |
| HDAC2 | 3 | 1256.41 |

**Supplementary Table 4-** Top ten genes from PPI of the HIF and DNMT family genes prioritized based on topological parameters.

| Putative miRNA targeting HIF family genes (Phase I) | | | | | | Global validation values (Phase II) | |
| --- | --- | --- | --- | --- | --- | --- | --- |
| Putative/DE miRNA | **Position in the UTR** | **seed match** | **Target genes from HIF gene family** | **conserved branch length** | **Pct** | **Combined Tstat/ Differential expression** | **Combined Pval** |
| hsa-miR-210 | 72-78 | 7mer-m8 | HIF3A | 2.93 | 0.27 | 128.68 | 0 |
| hsa-miR-483-3p | 2965-2972 | 8mer | HIF1A | 0.22 | N/A | 59.498 | 4.47E-06 |
| hsa-miR-361-3p | 535-541 | 7mer-m8 | EPAS1 | 0 | N/A | 55.075 | 1.88E-05 |
| hsa-miR-301b | 865-871 | 7mer-1A | HIF1A | 0.489 | N/A | 51.1 | 5.74E-05 |
| hsa-miR-342-3p | N/A | N/A | N/A | N/A | N/A | 46.993 | 2.50E-04 |
| hsa-miR-520d-3p | 1499-1505 | 7mer-m8 | EPAS1 | 4.331 | 0.7 | 46.166 | 2.98E-04 |
| hsa-miR-339-3p | N/A | N/A | N/A | N/A | N/A | 42.961 | 6.69E-04 |
| hsa-miR-574-3p | 51-57 | 7mer-m8 | HIF3A | 0.532 | N/A | 37.716 | 0.003927 |
| hsa-miR-128 | 42-48 | 7mer-1A | ANRT2 | 0.285 | < 0.1 | 37.586 | 0.003927 |
| hsa-miR-520h | 1501-1507 | 7mer-m8 | EPAS1 | 0.009 | N/A | 37.526 | 0.003927 |
| hsa-miR-519d | 1104-1110 | 7mer-m8 | HIF1A | 7.503 | 0.74 | 36.298 | 0.0051746 |
| hsa-miR-582-5p | 1273-1279 | 7mer-m8 | HIF1A | 5.753 | N/A | 35.7 | 0.0061153 |
| hsa-miR-107 | 953-960 | 8mer | ANRT | 3.506 | 0.69 | 34.798 | 0.0080929 |
| hsa-miR-193a-5p | 592-598 | 7mer-m8 | HIF3A | 0.029 | < 0.1 | 34.211 | 0.0095495 |
| hsa-miR-516a-5p | N/A | N/A | N/A | N/A | N/A | 33.268 | 0.012717 |
| hsa-miR-34c-5p | 829-835 | 7mer-1A | ANRT | 0.054 | N/A | 32.327 | 0.016476 |
| hsa-miR-501-5p | 343-349 | 7mer-m8 | EPAS1 | 0.149 | N/A | 32.068 | 0.01698 |
| hsa-miR-518b | N/A | N/A | N/A | N/A | N/A | 32.017 | 0.01698 |
| hsa-miR-887 | 167-173 | 7mer-m8 | HIF3A | 0 | N/A | 30.733 | 0.0248 |
| hsa-miR-28-5p | 1398-1404 | 7mer-m8 | -0.02 | 2.913 | 0.17 | 29.871 | 0.032319 |
| hsa-miR-125a-3p | 1140-1146 | 7mer-1A | ANRT2 | 0.613 | N/A | -30.783 | 0.0248 |
| hsa-miR-484 | 1860-1866 | 7mer-m8 | HIF3A | 0 | N/A | -33.168 | 0.012717 |
| hsa-miR-140-5p | 3731-3737 | 7mer-m8 | HIF1A | 2.407 | 0.38 | -36.369 | 0.0051746 |
| hsa-miR-125a-5p | 3250-3256 | 7mer-1A | ANRT2 | 2.535 | < 0.1 | -36.373 | 0.0051746 |
| hsa-miR-520b | 1499-1505 | 7mer-m8 | EPAS1 | 4.331 | 0.7 | -41.262 | 0.0011851 |
| hsa-miR-520f | 1475-1481 | 7mer-m8 | EPAS1 | 3.453 | 0.52 | -43.872 | 5.15E-04 |
| hsa-miR-372 | 1499-1505 | 7mer-m8 | EPAS1 | 4.331 | 0.7 | -44.681 | 4.17E-04 |
| hsa-miR-516a-3p | 2492-2499 | 8mer | HIF1A | 0.024 | N/A | -45.089 | 3.99E-04 |
| hsa-miR-520c-3p | 1499-1505 | 7mer-1A | EPAS1 | 4.331 | 0.7 | -52.649 | 3.82E-05 |

**Supplementary Table S5-** This table links the findings from Phase I where we generated a list of putative miRNAs associated with hypoxia; and these were validated in phase II using the publically available miRNA microarray datasets related to hypoxia. Apart from the miRNAs marked in red all other differentially expressed miRNAs (DE miRNAs) were found to be common between phase I and phase II of the analysis.
